# Experimental and hypothetical appraisal on inhibition of glucose-induced glycation of bovine serum albumin by quercetin

**DOI:** 10.1186/s43141-023-00588-5

**Published:** 2023-11-16

**Authors:** Babatunde Joseph Oso, Ige Olaoye, Olufunke Temiloluwa Oso

**Affiliations:** 1 Department of Biochemistry, McPherson University, Seriki Sotayo, Ogun State Nigeria; 2 Department of Obstetrics and Gynaecology, Ekiti State University Teaching Hospital, Ado-Ekiti, Nigeria

**Keywords:** Glycation, Bovine serum albumin, Amyloid beta-peptide, Quercetin, Molecular docking

## Abstract

**Background:**

The specificity of protein functions depends on its folding ability into a functional structure. Protein folding is an essential systemic phenomenon that prevents incorrect folding which could result in harmful aggregation. This harmful aggregation of proteins causes neurodegenerative diseases and systemic amyloidosis. Experimental and theoretical approaches were used in this study to explicate the probable mechanisms of action of quercetin in inhibition of glucose-induced glycation through estimations of percentage glycated protein, inhibited induced protein aggregation, and unoxidized bovine serum albumin thiol groups and assessments of molecular interactions of quercetin with the structures of bovine serum albumin, amyloid beta-peptide (1–42) and 3D amyloid-beta (1–42) fibrils retrieved from the protein databank (http://www.rcsb.org).

**Results:**

The results showed quercetin inhibited the formation of glycated protein, protein aggregation, and thiol oxidation in a concentration-dependent manner where 200 μg/ml showed the highest inhibition while 50 μg/ml depicted the least inhibition in all the studied assessments. From the docking analysis, it was observed that quercetin had a significantly higher binding affinities − 8.67 ± 0.09 kcal/mol, − 5.37 ± 0.05 kcal/mol and − 5.93 ± 0.13 kcal/mol for the bovine serum albumin, amyloid beta-peptide (1–42) and 3D amyloid-beta (1–42) fibrils respectively compared to the glucose, the inducer. Quercetin and glucose interacted with amino acid residues at the BSA subdomain IIA thus providing a clue that quercetin may impose its inhibition through the binding domain. Also, it is important to mention that the phytochemicals shared a similar interaction profile as that of glucose with the amyloid-beta.

**Conclusions:**

These findings established the beneficial effects of quercetin as a potential agent that could alleviate hyperglycaemic-initiated disorders associated with elevated serum glucose levels.

## Background

Quercetin is a flavonol belonging to a family member of flavonoids which are themselves part of a large class of polyphenols and exist in many plant parts commonly as glycosides. Its chemical structure which includes a pyrocatechol group, and a benzene ring allows it to act as an antioxidant [1]. It plays various roles in biological activities involved in the vegetative growth of botanicals. It is obtained through dietary sources in free aglycone form or conjugated with glycosides or methyl ethers at the hydroxyl groups [2]. Free quercetin easily gets absorbed through the colonic mucosa; the glycosylated form is prepared for absorption by the colonic mucosa through its hydrolyzation by β-glucosidases in the digestive tract [3]. Quercetin has been reported to play beneficial roles in the protection of macromolecules such as lipids, proteins, and nucleic acid against oxidation. Various biological properties, such as anti-oxidative, anti-inflammatory, anti-diabetic, and neuroprotective, have been associated with quercetin. Considerable evidence from experimental studies has shown that quercetin may exhibit protective effects against vascular diseases such as atherosclerosis and myocardial infarction, and systemic oxidative stress associated with glucotoxic conditions [4–7]. Moreover, it has been suggested through various experimental data that quercetin alleviates dysfunction of the arterial wall and vascular diseases characterized by thickening of the arterial wall through its vasodilating effects [8]. Hyperglycemia, a critical factor in diabetic complications, is a causal path which had been implicated in the pathogenesis of diseases associated with vascular dysfunction. A strong correlation has been established between the formation of serum advanced glycation end products (AGEs) which are products of non-enzymatic glycation and oxidation of nucleic acids, proteins, and lipids, and the development and progression of vascular diseases such as diabetic macro and microvascular complications (e.g., retinopathy, neuropathy, and nephropathy) associated with hyperglycemia [9, 10]. Also, the denaturation condition of proteins via glucose-induced glycation was studied using NMR characterization [11]. More so, quercetin through experimental data has been shown to inhibit the glycation of these biological molecules [12]. Moreover, quercetin has been shown to exhibit good absorption, distribution, metabolism, and drug-likeness properties without any toxic effects [13]. Presently, there is a paucity of studies on the mechanism of the antiglycation effect of quercetin. Thus, this current study aimed to investigate the mechanism of inhibitory actions of quercetin on non-enzymatic glucose-induced glycation of bovine serum albumin in various mechanistic studies.

## Methods

### Chemicals

All chemicals used were of analytical grade. Bovine serum albumin (BSA) and Congo red are products of Kem Light, India. Methanol, sodium dihydrogen phosphate, and disodium hydrogen phosphate were products of Guangzhou JHD Chemical Reagent Co., Ltd., Guangzhou, China.

### In vitro glycation of bovine serum albumin

The procedure for the preparation of glycated albumin was according to the method of Safari et al. [14] with minor modifications. Briefly, BSA (0.15 g/ml) was incubated in phosphate buffer (0.1 molar, pH = 7.4, containing 0.01% sodium azide) containing D-glucose (18 mg/ml) as a glycated group. The control group had the same concentration of BSA prepared in phosphate buffer (0.15 g/ml) without D-glucose. To study the influence of quercetin on BSA glycation, different concentrations of quercetin (50, 100, 150, and 200 μg/ml) freshly prepared in methanol were added to the incubation medium containing BSA (0.15 g/ml) and D-glucose (18 mg/ml) in phosphate buffer (0.1 molar, pH = 7.4, containing 0.01% sodium azide). The solutions were incubated at 37 °C for 72 h. The reaction mixtures were then dialyzed against phosphate buffer (0.01 molar, pH = 7.4) for 24 h to remove unbound glucose from the samples.

### Measurement of glycated BSA

The degree of BSA glycation was estimated through the colorimetric method using thiobarbituric acid (TBA) [15, 16]. In principle, glycated BSA is hydrolyzed by oxalic acid to produce 5-hydroxymethyl furfural (5-HMF) which reacts with TBA to form a chromophore with a maximum absorbance at 443 nm. Briefly, 2 ml of 20% trichloroacetic acid (TCA) was added to 4 ml of glycated sample and centrifuged for 10 min at 600×*g*. Exactly 1 ml phosphate buffer and 0.5 ml oxalic acid (0.3 N) was added to the sediment and boiled in the water bath for 1 h. After cooling, 0.5 ml of 40% TCA was added to each sample and centrifuged (10 min, 3000 rpm). 0.5 ml 0.05 molar TBA was then added to 1 ml of supernatant and heated to 40 °C for 30 min. The absorbance of each sample was taken at 443 nm. The results were reported as the percentage inhibition of protein glycation.

### Determination of protein aggregation

Congo red was used to assess the effect of quercetin on glycation-induced BSA aggregation [17]. Congo red binds to amyloid-beta and forms a complex with maximum absorbance at 530 nm. Briefly, 0.5 ml of glycated BSA was incubated with 0.5 ml of Congo red (100 μmolar in phosphate buffer with 10% ethanol) at 25 °C for 20 min. The absorbance of the sample was then recorded at 530 nm. The results were reported as the percentage Inhibition of protein aggregation.

### Determination of inhibition of glycation-induced oxidation of protein thiol groups

The determination was carried out as described by Ellman [18]. Precisely, 1.0 ml of 0.5 mmolar 5, 5′-dithiobis (2-nitrobenzoic acid) in 0.1 molar phosphate buffer (pH 7.4) was added to a test tube containing 1.0 mL of the glycated sample and incubated at room temperature of 29 °C for 15 min. The absorbance was measured at 412 nm. The results were reported as the percentage inhibition of thiol oxidation.

### Molecular docking

The 3D SDF structures of the selected quercetin (PubChem CID: 5280343), and glucose (PubChem CID: 5793) were obtained from the PubChem database (http://www.pubchem.ncbi.nlm.nih.gov) [19]. The structures of bovine serum albumin, amyloid beta-peptide (1–42), and 3D amyloid-beta (1–42) fibrils with PDB IDs 4OR0, 1IYT and 2BEG were retrieved from the protein databank (http://www.rcsb.org) [20]. The molecules were subsequently converted to the pdbqt format using Autodock tools. Blind docking of the ligands to various protein targets and determination of binding affinities was carried out using PyRx-Python Prescription 0.8 (The Scripps Research Institute) [21] with the grid st at (center: *X* 35.1650; *Y* 23.9873; *z* 98.0448), (*X* − 0.6159; *Y* − 0.0652; *Z* 11.2694), and (*X* 0.4623; *Y* 0.2758; *Z* − 8.9351), respectively with dimension: dimension: (*X* 144.9461; *Y* 64.2626; *Z* 88.7264), (*X* 33.6613; *Y* 22.3151; *Z* 49.5333), and (*X* 45.8883; *Y* 22.9133; *Z* 27.2084). The interactions were visualized through BIOVIA. The binding affinities of compounds for the three protein targets were recorded.

### Molecular dynamics of interaction quercetin and glucose with BSA

WebGRO Protein with Ligand Simulation (http://simlab.uams.edu/ProteinWithLigand/protein_with_ligand.html) was used for the molecular dynamic study [22]. The **ligand topology** files were generated from PRODRG using the GlycoBioChem PRODRG2 Server (http://davapc1.bioch.dundee.ac.uk/cgi-bin/prodrg/) with the temperature set at 300 K, pressure set at 1.0 bar, simulation time set at 1 ns, and an approximate number of frame per simulation was set at 1000 while Leap-Frog was chosen as the molecular dynamics integrator [23]. Other settings were maintained as default using GROMOS96 43a1 as a Force field. The simulation results computed are the root mean square deviation of the given structure over time (RMSD), root mean square fluctuation of each residue in the given structure (RMSF), the radius of gyration or structural compactness (Rg), solvent-accessible surface area (SASA), and average number of H-bonds in each frame over time (H-bonds).

### Statistical analysis

The findings were evaluated using one-way variance analysis (ANOVA) for mean differences between the different concentrations of quercetin followed by multiple comparison tests by Duncan for post-hoc correlations at *p* < 0.05 and reported as a means ± standard deviation of three determinations.

## Results

The inhibition of protein glycation, protein aggregation, and thiol oxidation were assessed to measure the availability of free unoxidized proteins for their biological functions. The result of the study revealed a significant decrease in the glycated BSA in a quercetin concentration-dependent manner. Similarly, a dose-dependent significant decrease in protein aggregation was observed where the highest quercetin concentration (200 μg/ml) showed the highest percentage of protein aggregation inhibition (66.18 ± 0.91%). In addition, the highest thiol oxidation percentage inhibition (78.51 ± 2.58%) was seen in the highest quercetin concentration (Table 1).

**Table 1:** Percentage inhibitory effects of quercetin on bovine serum albumin glycation, aggregation, and thiol oxidation

	200 μg/ml	150 μg/ml	100 μg/ml	50 μg/ml
Inhibition of glycation (%)	76.69 ± 1.12^a^	64.96 ± 1.40^b^	57.40 ± 1.07^c^	49.75 ± 0.65^d^
Inhibition of aggregation (%)	66.18 ± 0.91^a^	36.10 ± 1.55^b^	36.46 ± 2.55^c^	32.98 ± 4.34^d^
Inhibition of thiol oxidation (%)	78.51 ± 2.58^a^	74.22 ± 1.66^b^	60.81 ± 10.69^c^	47.96 ± 1.26^d^

Different superscripts across the row indicate significant differences at *p* < 0.05

**Fig. 1:**
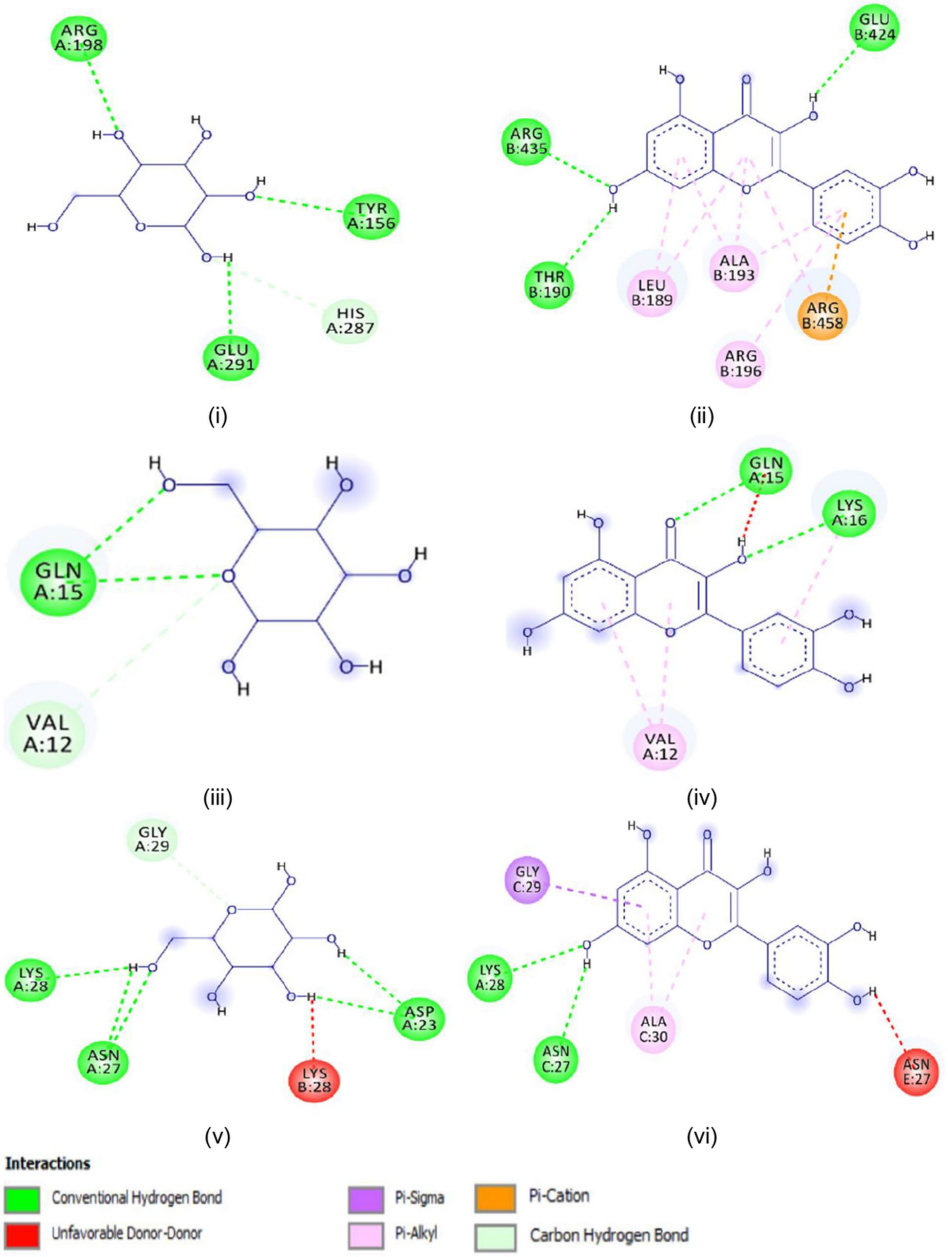
2D interactions of **i** glucose and **ii** quercetin with the structures of **a** bovine serum albumin, **b** amyloid beta-peptide (1–42), and **c** amyloid-beta (1–42) fibrils

**Fig. 2:**
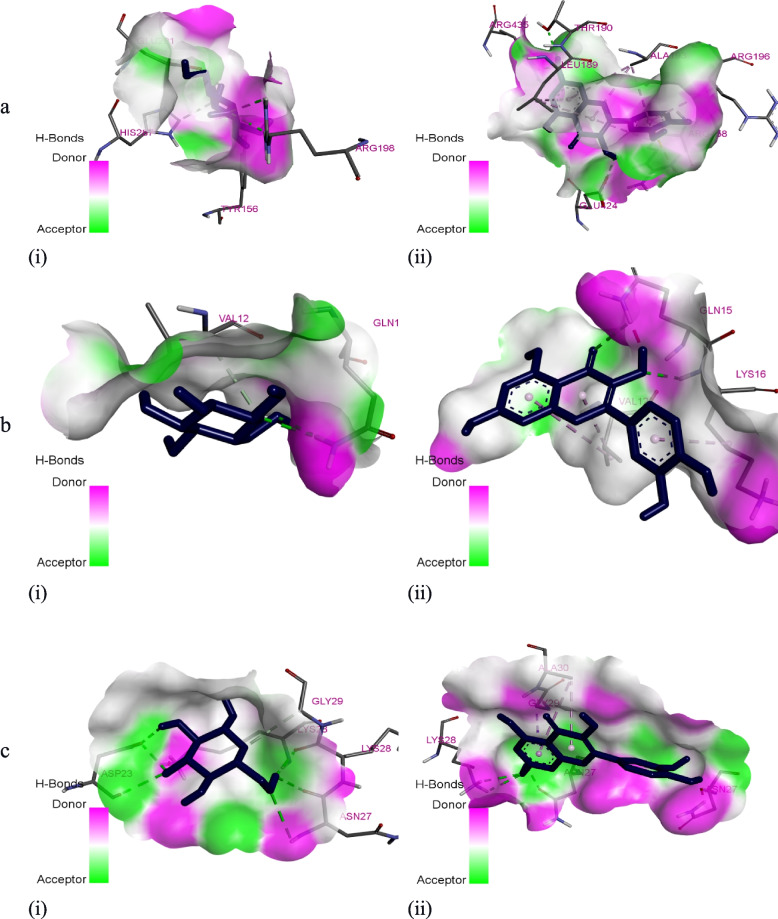
3D interactions of **i** glucose and **ii** quercetin with the structures of **a** bovine serum albumin, **b** amyloid beta-peptide (1–42), and **c** amyloid-beta (1–42) fibrils

**Fig. 3:**
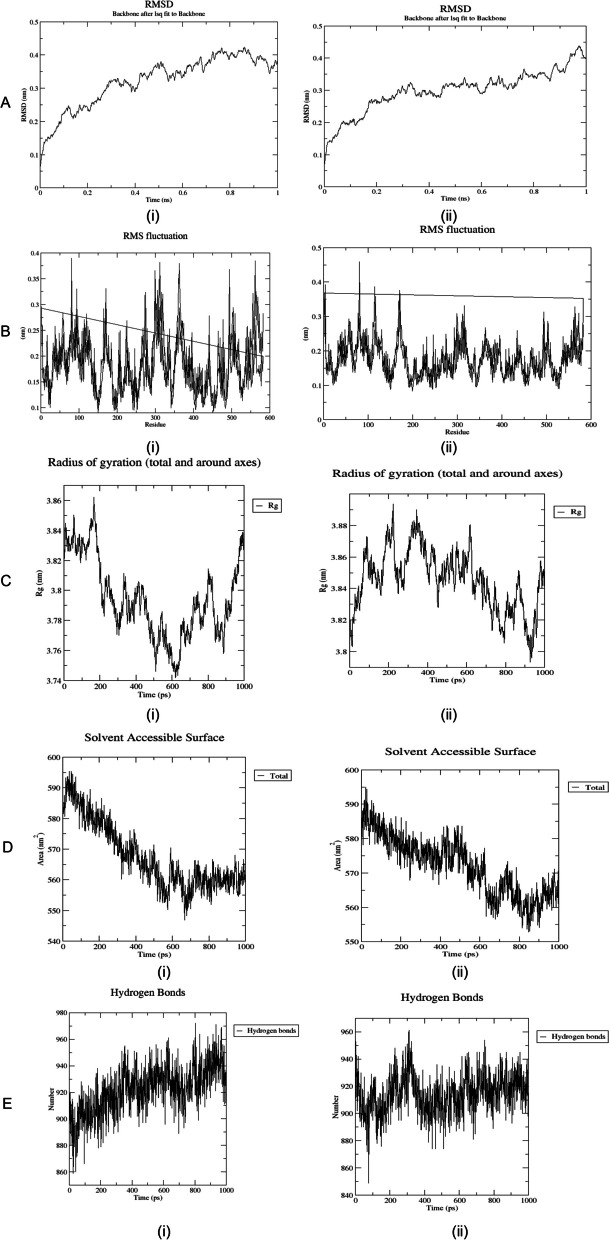
Molecular dynamics simulation parameters showing the **A** root mean square deviation (RMSD), **B** root mean squared fluctuations (RMSF), **C** radius of gyration (Rg), **D** solvent accessible surface area (SASA), and **E** H-bonding of BSA docked with **i** glucose and **ii** quercetin

The in vitro work was substantiated with the computational analysis to give a clear molecular understanding of quercetin inhibition potential on glycation, protein aggregation, and thiol oxidation. The result depicted that in all the proteins (BSA, amyloid beta peptide (1–42), and amyloid beta (1–42) fibrils) examined, quercetin showed a significant higher binding scores (− 8.67 ± 0.09, 5.37 ± 0.05, and 5.93 ± 0.13 kcal/mol respectively) than glucose (Table 2). Also, different binding residues were observed for BSA from glucose and quercetin suggesting different binding sites for glucose and quercetin. Meanwhile, similar interacting residues are seen in amyloid beta peptide (1–42) (Val12 and Gln15), and amyloid beta (1–42) fibrils (Asn27 and Lys28) for glucose and quercetin suggested the same binding sites for glucose and quercetin (Figs. 1 and 2). Furthermore, the dynamics simulation was carried out to evaluate the influence of quercetin and glucose on the stability of the studied protein. It is evident from Fig. 3 that the molecular dynamics results showed higher root mean square of fluctuation (RMSF), radius of gyration (Rg), and solvent-accessible solvation area (SASA) for BSA-quercetin complex than BSA-glucose complex. However, a lesser root mean square of deviation occurred in the BSA-quercetin complex when compared to the BSA-glucose complex.

**Table 2:** Binding affinities in kcal/mol and identified interaction sites of glucose and quercetin with bovine serum albumin, amyloid beta-peptide (1–42), and amyloid-beta (1–42) fibrils

		Glucose	Quercetin
Bovine serum albumin	Binding score (kcal/mol)	− 5.63 ± 0.05^a^	− 8.67 ± 0.09^b^
	Hydrogen bond	Tyr156, Arg198, Glu291	Thr190, Glu424, Arg435
	Pi-Alkyl		Leu189, Ala193, Arg196
	Pi Cation		Arg458
Amyloid beta-peptide (1–42)	Binding score (kcal/mol)	− 3.77 ± 0.05^a^	− 5.37 ± 0.05^b^
	Hydrogen bond	Val12, Gln15	Gln15, Lys16
	Pi-Alkyl		Val12
Amyloid beta (1–42) fibrils Pi-sigma	Binding score (kcal/mol)	− 4.57 ± 0.05^a^	− 5.93 ± 0.13^b^
	Hydrogen bond	Asp23, Asn27, Lys28	Asn27, Lys28
	Pi-Alkyl		Ala30
			Gly29

Different superscript in the row indicates significant differences at *p* < 0.05

## Discussion

This study investigated the probable protective effects of quercetin on the amelioration of secondary complications associated with hyperglycemia and the formation of advanced glycated end products. The study was carried out by initially assessing the direct protective effect of quercetin on the non-enzymatic glycation of BSA outside the living system. Data obtained for the antiglycation indices (Table 1) shows incubation of varying concentrations of quercetin (50, 100, 150, and 200 μg/ml) with the mixture of BSA (0.15 g/ml) and D-glucose (18 mg/ml) significantly reduced (*p* < 0.05) the percentage of glycated BSA in a concentration-dependent manner. A dose-dependent decrease in the percentage of glycation-induced protein fibrillation was also noted in all the samples with quercetins. Furthermore, there was a considerably greater reduction in the levels of glycation-induced thiol oxidation in the samples incubated with different concentrations of quercetin. Significant reductions (*p* < 0.05) were observed at all the concentrations. The results showed that quercetin protects against glucose-induced BSA glycation. Quercetin limited the reaction of biological amines of the BSA by reducing ends of glucose thus preventing the formation of glycated albumins. This result correlates with the studies of Li et al. [24] and Alam et al. [25] where quercetin inhibited the glycation of BSA and human serum albumin respectively.

Excessive accumulations of glucose and reactive sugar derivatives in the serum have been implicated in the pathogenesis of severe and chronic diseases characterized by vascular damage and oxidative stress. Elevated serum level of AGEs formed through the Maillard reaction has been reported as a biomarker for coronary artery atherosclerosis in patients with diabetes mellitus [26]. Maillard process involves the non-enzymatic glycation of amino acid residues of proteins leading to the formation of Schiff base, an unstable compound that undergoes molecular rearrangements forming the Amadori product, a more stable compound. The Amadori product undergoes a series of oxidation to form a more stable compound known as the advanced glycation end product (AGE). The formation of the AGE in vascular wall collagen could trigger crosslinking of the collagen molecule. This leads to thickening of the basement membrane, plaque formation, development of unstable peptide plaques, and loss of vascular elasticity. Diseases that are associated with uncontrollable protein glycation and induced protein fibrillation include neurodegenerative disorders, such as amyotrophic lateral sclerosis and Alzheimer’s disease, atherosclerosis, and diabetic microvascular complications such as retinopathy, neuropathy and nephropathy and [27–29]. Quercetin has been reported to possess various biological benefits, such as antibacterial, anti-inflammation, immune-modulatory, and the ability to inhibit fibril-induced aggregation of proteins [30–32]. These biological properties are exceedingly valuable in the prevention and management of these disorders that are associated with complications of diabetes mellitus which include cardiovascular diseases by halting the amplification loop leading to the sustenance of AGE generation, fibril formation, induced oxidant stress, and vascular damage. This shows the compound could prevent AGEs-induced exaggerated secondary complications associated with hyperglycemia such as vascular dysfunctions characterized by increased vasoconstriction and impaired vasodilation. This outcome is in relation to previously reported claims that quercetin administration improved vasoconstriction and enhanced vasodilation in experimental animals with hyperglycaemic-induced vascular diseases [33, 34]. Moreover, these secondary complications of diabetes mellitus have also been hypothesized to be induced by the oxidation of serum proteins [35]. The antioxidative effect of quercetin on associated induction of thiol oxidation investigated through Elman’s reagent strongly established quercetin as possessing antioxidant benefit by protecting biological molecules such as proteins, lipids, and nucleic acids against oxidation and maintaining systemic redox balance.

### Molecular docking

The obtained results of the in vitro study were supported by the molecular study to understand the inhibitions mechanisms/interactions of quercetin towards a complex transporter (BSA), amyloid beta-peptides, and the partially not paired beta-strands fibrillary ends (amyloid-beta fibrils) (Table 2). The interactions of glucose and quercetin towards BSA occurred in the BSA subdomain IIA known as the site I though with different residues. (Figs. 1 and 2) [36, 37]. In addition, both ligands formed three equal numbers of hydrogen bonds with the interacting residues where bonding electrons of two bonds are donated from the glucose –OH to BSA residues: Tyr156 and Arg198 while one from the oxygen of BSA Glu291 residue. However, the bonding electrons of two bonds are donated from the oxygen of BSA Thr190 and Glu424 residues to the –OH of quercetin while the –OH group of quercetin donated the last hydrogen bonding electrons to the Arg435. This equal number of conventional hydrogen bond formation might not be responsible for the differences in their binding scores (Table 2) as suggested in the work of Elokely and Doerksen [38] but could be due to the number of pi-interactions such as pi-cation and pi-alkyl interactions that occurred between quercetin and BSA residues like Leu189, Ala193, Arg196, and Arg458 [39]. The higher binding affinity due to a higher binding score (− 8.67 ± 0.09 kcal/mol) observed in quercetin agreed with the finding of Shinde et al. [40] on amiloride-BSA interaction where quercetin produced a higher binding constant than amiloride in BSA site II. 

Similarly, quercetin has a better binding score (− 5.37 ± 0.05 kcal/mol) (Table 2) than glucose with amyloid beta-peptide (1–42) with an equal number of conventional hydrogen bonds with Gln15 and Lys16 for quercetin while Gln15 only for glucose might contribute to the higher binding score in quercetin but the pi-interaction like pi-alkyl (Figs. 1 and 2) could be the favorable factor [38, 39]. The molecular docking results of the two ligands showed different affinities with amyloid-beta fibril in the same binding region of the molecule (Figs. 1 and 2). However, the potency of quercetin was pronounced through the higher binding score compared to glucose (Table 2). None of the ligands binds to the tip, i.e., a probable region (residues 17–21) of the amyloid-beta fibril for the addition of incoming amyloid beta monomer [41]. However, both ligands interacted at two of the suggested distinct regions of amyloid-beta fibril: a turn region formed with one of the residues (Asn27) and the second hydrophobic region with two residues: Gly29 and Ala30 (Figs. 1 and 2) [42]. The interaction at these two regions could prevent the aggregation of amyloid-beta monomers with quercetin possessing more probability of amyloid-beta fibril inhibition due to its higher binding score (− 5.93 ± 0.13 kcal/mol). Previous studies on the effects of glycation on beta amyloids have confirmed that glycation by glucose increases the rate of self-assembly and aggregate size of beta amyloids [43, 44]. The computed increased affinity of quercetin for the peptide could inhibit glucose-induced self-assembly of amyloid aggregates and the formation of the fibrillary aggregates.

### Molecular dynamics studies for BSA docked with glucose and quercetin

A molecular dynamics simulation was performed to understand the glucose and quercetin-induced dynamical behavior in the conformational space of BSA. The dynamics obtained evaluated the stability and changes of the BSA secondary structure. The parameters analyzed to define the stability of the BSA–ligand complexes are the root mean square deviation (RMSD), root mean squared fluctuations (RMSF), radius of gyration (Rg), solvent accessible surface area (SASA), and H-bond variations. The generated coordinates of the trajectories drawn for the specified period of the simulation are presented in Fig. 3. The trajectories indicate distinct differences and variations in the dynamic motions of the components of BSA required for biological function between the two complexes [45]. The RMSD which is the measure of the average distance between the atoms (usually the backbone atoms) revealed the structural stabilities of BSA complexes with glucose and quercetin. Glucose-induced greater change in the RMSD of BSA backbone stability as shown by the trajectory with a higher degree of deviation compared to quercetin. This implies a greater significant conformational change could occur in BSA when bound with glucose. The trajectory of the measure of the induced deviation between the atomic positions of each amino acid of BSA and the sensitivity of different residues towards the ligands computed through the RMSF revealed quercetin greatly induced fluctuation in the conformational space of BSA. More so, changes in the conformational stability in term of the strength of the BSA-ligand system was explored by studying the Rg of the BSA-ligand complexes. Rg estimates the distance between the axis of rotation and the center of mass [46]. Glucose-induced lesser fluctuations in Rg values compared to quercetin; this indicates more conformational stability and a compact system of the BSA-glucose complex. Further simulation on the solvent accessible surface area (SASA) on the available BSA binding sites revealed BSA-quercetin complex had larger SASA which implies better favorable binding of quercetin with BSA. However, the simulated amounts of hydrogen bonds formed between the amino acid residues in the BSA-ligand complexes were greater in the complex formed by binding with glucose. This shows greater stability of the BSA-glucose complex. The obtained trajectories of the molecular dynamic simulation theoretical analysis to understand and compare the induced conformational changes upon binding of glucose and quercetin presumed that the BSA-quercetin complex had more dynamic fluctuations perhaps caused by the induced enhancement of flexibility of the BSA binding site.

## Conclusions

In the present study, quercetin was found to inhibit the glycation of BSA, limit protein aggregation, and inhibit the oxidation of BSA thiol groups induced by elevated glucose concentration in a concentration-dependent manner. The observations show that the compound has the potential to interact with amino acid residues of serum albumin. These findings established the beneficial effects of quercetin as a potential agent that could alleviate hyperglycaemic-initiated vascular damage. It also suggests that inhibition of the formation of AGEs, prevention of protein fibrillation, and oxidation of protein thiol could be a common probable mechanism for the vascular protective effect of quercetin. This insight could have ever-changing effects on the development of biological agents for the management of diseases that are associated with hyperglycemia. However, further works are encouraged for the recommendation of its prospective use as an alternative therapeutic agent against disorders of hyperglycemia.

## Data Availability

The dataset supporting the conclusions of this article is included as tables and figures in the manuscript.

## Abbreviations

3D: Three dimensional
AGEs: Advanced glycation end products
BSA: Bovine serum albumin
TBA: Thiobarbituric acid
TCA: Trichloroacetic acid
RMSD: Root mean square deviation
RMSF: Root mean square fluctuation
Rg: Radius of gyration
SASA: Solvent accessible surface area
ANOVA: One-way variance analysis
